# Comparison of Resistant and Susceptible Strains of *Trichomons vaginalis* to Metronidazole Using PCR Method

**Published:** 2012

**Authors:** S Rabiee, A Bazmani, M Matini, M Fallah

**Affiliations:** 1 Dept. of Obstetrics and Gynecology, Fatemieh Women Hospital, Hamadan University of Medical Science, Iran; 2 Drug Applied Research Center, Tabriz University of Medical Sciences, Tabriz, Iran; 3 Dept. of Parasitology and Mycology, School of Medicine, Hamadan University of Medical Science, Hamadan, Iran; 4 Dept. of Parasitology and Mycology, School of Public Health, Tehran University of Medical Science, Tehran, Iran

**Keywords:** *Trichomonas vaginalis*, Metronidazole, Resistance, PCR

## Abstract

**Background:**

Metronidazole is drug of choice recommended by WHO for treatment of trichomoniasis, however, some reports claims drug resistance in *Trichomonas vaginalis* isolates recently. The objective of this study was to determine the minimum lethal concentration (MLC) of metronidazole in resistant and sensitive strains, as well as genetic patterns of these stains by PCR method.

**Methods:**

From February 2006 to March 2007, in a cross sectional study, clinical and wet mount examination of vaginal smear along with culture were performed on 683 women attending to public and private outpatient clinics in Hamadan. Trichomoniasis marked based on major clinical symptoms. Diagnosis confirmed using wet mount microscopically and culture in Diamond medium. A serial concentration of metronidazole was provided and all isolated *Trichomonas* strains (resistant and sensitive) tested by standard method. Finally, all sensitive and resistant strains examined by PCR technique.

**Results:**

Only 15/683, (2.2%) of patients clinically diagnosed trichomonal vaginitis were positive for *T. vaginalis* by wet smear and culture. The minimum lethal concentration (MLC) for clinically sensitive isolates was 25 µg/ml; however, this concentration for resistant isolates was 200 µg/ml after 24 h and 100 µg/ml after 50 h. The results of PCR examination of DNA from sensitive and resistant isolates had same pattern. The lanes appeared by two primers were 98 bp and 261 bp for both clinically sensitive and resistant strains.

**Conclusion:**

Resistance to metronidazole in *T. vaginalis* has not relation to genetic variations and might be related to some physiologic pathways of organism.

## Introduction

Trichomoniasis is recognized as an important sexually transmitted disease (STD) in the world and has high prevalence and incidence between all STD, even in the developed countries such as US. Its prevalence strongly related to various cultural and social norms in different societies, in relation to sexual partnership, monogamy or polygamy etc. Metronidazole is an antimicrobial agent that has been used for more than 50 years for the treatment of infection caused by *Trichomonas vaginalis* and also other important intestinal protozoa, such as *Giardia lamblia* and *Entamoeba histolytica* (1). Metronidazole is a nitroimidazole derivative which was primarily developed to treat *Trichomonas* infections but gradually it became a drug of choice for the treatment of infections caused by *Entamoeba*, *Giardia*, and anaerobic bacteria recommended by various pharmacopeia. This agent is also useful in the treatment of systemic anaerobic infections, such as infections caused by *Bacteroides fragilis*, *Clostridium difficile* and so on (2, 3). Treatment regimens administered for the eradication of *Helicobacter pylori* in the gastro-duodenitis patients still include metronidazole in combination with other antibiotics. In spite of 50 years of extensive use of this drug, metronidazole still remains the favorable standard agent for the treatment and prophylaxis of infections caused by anaerobic agents.

Metronidazole more often produces good clinical cure when it is used for treatment of trichomoniasis, giardiasis, and amoebiasis in a standard recommended dose, and also it is recommended for the treatment of patients with bacterial vaginosis or nonspecific vaginitis caused by *Gardnerella vaginalis* (4).

In spite of many beneficial effect of metronidazole, there are increasing reports of treatment failure clinically in trichomoniasis (5–8).

According to some reports, metronidazole resistance is a major problem in clinical isolates of *T*. *vaginalis* in the United States and elsewhere, while metronidazole-resistant *Entamoeba* and *Giardia* have for the most been prepared by selection in culture (9). There is no report about the metronidazole resistance of *T. vaginalis* in Iran; however, some observations indicate refractory cases of patients clinically.

The main goal of the present study was to determine minimum inhibitory concentration (MIC) of metronidazole against *T. vaginalis*, in vitro, isolated from Iranian patients and, study of genetic profile of resistant and sensitive strains by PCR technique.

## Materials and Methods

The target population of this study was the patients admitted to gynecology clinics in Hamadan having the vaginal disorders suspected to vaginal trichomoniasis. All patients went under clinical examination and two vaginal specimens (with cotton swab) were taken from all with vaginal infection signs. Primarily, direct wet mount examination was done and another specimen was cultured in Diamond medium for *T. vaginalis*. The patients confirmed with trichomoniasis received one standard dose of metronidazole (2g single dose) and treatment repeated one more dose if the complaints were continued and parasite were detected again by same methods. Resistant cases defined as “cases in which two standard courses of treatment fails to cure clinically and parasite detect by above-mentioned methods” (6).

Minimum lethal concentration (MLC) of metronidazole (Sigma Chemical Co. St. Louis) was done for all isolated parasites by method described by Upcroft and Upcroft, 2001 (4). *T. vaginalis* isolates (n = 10), susceptible to metronidazole clinically, and five drug-resistant isolates of parasite were used in this study. These wild-type strains were isolated from female patients examined at the University Women Hospital and in a private clinic. The symptomatic infection of most patients was successfully cured with a single course of metronidazole and drug susceptibility of the isolated strains was confirmed by an in vitro assay for MLC test.

### Drug susceptibility test

All parasites isolated from symptomatic patients were grown in culture medium. The parasites were sub-cultured three times a week in the Research Laboratory of Department of Parasitology & Mycology. Parasites to be used in drug susceptibility assays were grown for 1 day more following regular sub-culturing and were in the log phase of growth.

This experiment was done in the condition of aerobic assay. Stock solution (0.1 M) of drug (200 mg/ml metronidazole [Sigma]) in dimethyl sulfoxide (Sigma) were prepared and stored at -20°C until use. Metronidazole purchased in powder stock and was dissolved in DMSO 100%; drug went into solution in DMSO difficulty. To obtain better solubility, solution was autoclaved at 121° C for 15 min at15 lb/in^2^ (10).

The stock solution was diluted in medium to the research required concentrations. A useful starting concentration was 400µM, which yielded a maximum concentration in the assay of 100µM (20µg of metronidazole per ml).

Drugs were serially diluted, with dilutions ranging from 400 µg/ml to 3µg/ml. Plates were then incubated at 37°C for 48 h and read with an inverted phase-contrast microscope (Olympus, Japan). The micro plate titration well with the lowest concentration of drug in which no motile trophozoites were seen was reported as the MLC. The end point was confirmed by failure of non-motile parasites to grow after re-inoculation into drug-free medium.

For each isolate and drug concentration, there were four replicates with drug and two without drug. In this study, for *T. vaginalis* cultures 10x5 trophozoites per well were required for aerobic assays.

According to Upcroft and Upcroft method (2001), the plate aerobic assay mixtures were placed directly in a non-gassed incubator. Trophozoite growth was monitored daily by comparing control and drug-containing wells in the same row using an inverted microscope. Trophozoite numbers were scored using 1+ (dead or significantly fewer [not more than 20% coverage of total well surface] and significantly less active, 2+ (20 to 50% coverage of the total well surface and some parasite motility), 3+ (an almost confluent well, much motility), and 4+ (a confluent well) (11).

### PCR assay

Genomic DNA was extracted from specimens by phenol-chloroform-isoamyl alcohol method. A PCR assay was done by following primers (12):

TVK3: AT TGT CGA ACA TTG GTC TTA CCC TC, and TVK7: TCT GTG CCG TCT TCA AGT ATG C

These primers were used to amplify a 261-bp sequence of a *T. vaginalis*–specific repeat DNA fragment. The confirmatory assay used primers TVA5-1: AT GTT CTA TCT TTT CAT TGT and TVA6: GAT CAC CAC CTT AGT TTA CA to amplify a 98-bp fragment of a *T. vaginalis* specific sequence (12). PCR performed as described earlier (12). A peak on the gel electrophoresis corresponding to either a 261-bp or a 98-bp fragment was considered a positive PCR result, both in the resistant or sensitive isolates.

## Results

**Fig. 1:**
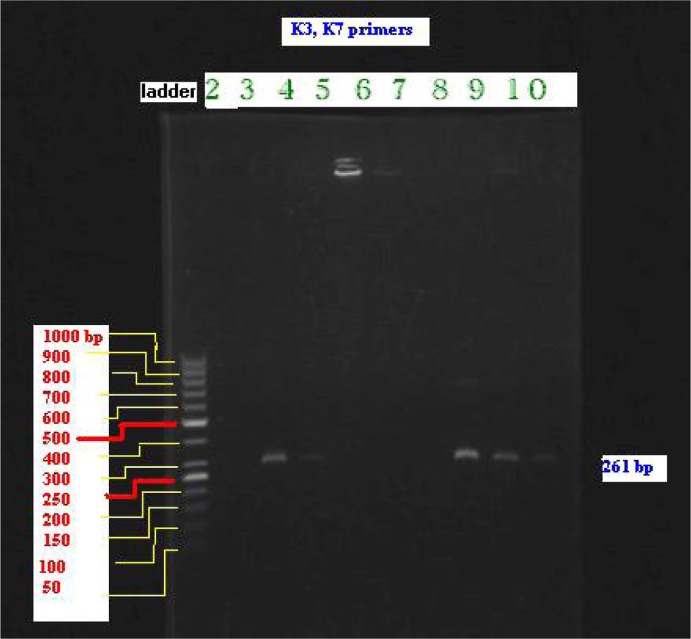
Electrophoresis of PCR product from clinically resistant and susceptible isolates of *T.vaginalis* with TVK3 and TVK7 primers on agarose gel. Lane 1 (far left) 1-kb DNA ladder (size marker); lanes 3-4 sensitive isolates (261bp); lanes 8-10 clinically resistant isolates. The expected product size was 261-bp. The gel shows same pattern for both isolates

A total of 683 patients attended to private outpatient clinics examined clinically and wet mount examination of vaginal smears along with culture were performed. A total of 15 women (2.2%) were found infected by *T. vaginalis*. Only 5 patients were found resistant to metronidazole clinically according to resistance criterion defined in this investigation. The MLC in the resistant isolates after 24 h incubation was 200 µg/ml, however, this concentration for sensitive isolates was 25 µg/ml; and after 50 h these concentrations were 100 µg/ml and 25 µg/ml respectively. Although both isolates, resistant and sensitive clinically, showed same pattern of bands in the PCR product (Fig. 1). Accordingly, the lane appeared by TVK7 primer were same in the resistant isolates (lane 3 and lane 4 related to sensitive and lanes 8, 9 and 10 related to resistant isolates with 261-bp respectively) had same pattern. In the other hand, the lane appeared for TVK3 primer had same pattern in the sensitive and resistant isolates at the 96-bp level.

## Discussion

In the present study, different concentrations of metronidazole on clinically susceptible and resistant isolates of *T. vaginalis* were shown for the first time in Iran. All isolates investigated, including metronidazole-resistant isolates, were susceptible to metronidazole, with different concentrations. The lowest effective concentration of metronidazole was 20µg/ml; however, the susceptibility of the isolates was dependent on exposed time. No significant differences in metronidazole susceptibilities were observed between metronidazole-susceptible and metronidazole-resistant isolates.

The mechanisms of drug resistance in parasites, especially in the protozoan parasites, has not explained clearly until know. Several mechanisms of resistance to metronidazole in anaerobic bacteria and intestinal protozoa have been proposed (13–15). These mechanisms differ among organisms, but the primary basis for resistance is “decreased uptake of the drug or altered reduction process efficiency”. These tow mechanisms act together; decreased activity of the nitroreductase leads to decreased uptake of the drug. Other mechanisms include active efflux, inactivation of the drug, and increased DNA damage repair (16). Specific resistance genes *(nim)* conferring resistance to nitroimidazoles have been isolated in different genera of gram-positive and gram-negative anaerobic bacteria, including *Bacteroides* species (17, 18). Some workers have been assigned a role in the expression of several resistance genes in *Bacteroides* species, including those for metronidazole, erythromycin-clindamycin, cef-oxitin, and carbapenems. Some workers expressed these elements can be found on the bacterial chromosome, on plasmids, and in multiple copies (19).

It has been proposed that clinical cases of drug-resistant *T. vaginalis* occur as aerobic resistance because O2 is required in some way to detoxify metronidazole; resistance has been accounted for as a decreased affinity for O2 by the respiratory system (15).

Despite the low levels of resistance to metronidazole in trichomoniasis, treatment failures attributed to metronidazole resistance have been reported during recent years (6). Although treatment failures are not uncommon but they have not yet clearly and completely attributed to drug-resistant strains. In a 10-year prospective study of isolates from patients who experienced treatment failure, all isolates had an MIC of <1 mg/L; thus, a decreased susceptibility of the infecting *C. difficile* strains was not considered to be the cause of the failures (3).

Treatment failure occurs in 5 to 20% of *Giardia* infections (4), and repeated treatment may be necessary. Resistance to metronidazole in the *H. pylori* stains have been reported that, combination therapy metronidazole with at least three antibiotics is recommended for eradication of organism in the cases of gastroduodenal ulcers. However, combination therapy is not recommended for refractory cases of clinical trichomoniasis until now. Drug resistance in *T. vaginalis* reported from 1962, but this phenomenon has not been as a major problem in clinical medicine and no appeared as an epidemic till now. Some investigators assumed that, resistance in the parasite may debilitates it and impede its transmission among sexually active partners, producing the resistance as a problem for the carriers alone. This assumption that the increased recognition of resistance to metronidazole in the *T. vaginalis* reflects an increased incidence or this is simply increased recognition of problem already is a case and needs more investigations along with need an alternative therapeutic to metronidazole as well.

Although, metronidazole is used yet as a cost-effective and almost safe drug in clinical medicine because of its cost, activity against wide spectrum anaerobic bacteria, favorable pharmaco-dynamic and pharmoco-kinetics and minor side effects (18).

*T. vaginalis* usually is not a major problem in Iranian ordinary patients, because the low prevalence according to reports based on the routine laboratory examinations (20–22); however, some workers detected higher prevalence of infection by PCR method (23) or in some high risk populations (24). Kazemi suggested mutation in ITS1 fragment of *T. vaginalis* in two (3.9%) of Iranian isolates which may be related to metronidazole resistance (25). Although, another report suggested some genetic diversity in different isolates of *T. vaginalis* from high risk population in Iran but, did not present any resistance evidence in the isolates (26, 27). Increases in the both prevalence of metronidazole–resistant *T.vaginalis* infection and the degree of resistance in the parasite indicate a need for a non-nitroimidazole treatment for refractory trichomoniasis, such as miltefosine (28). The current protocol for treating metronidazole–resistant trichomoniasis infections is increasing the dose of metronidazole and/or prescribes multiple doses regimen. Therefore, in spite of some difficulties in the treatment of trichomoniasis at present time, although drug resistance was first reported in 1962, metronidazole is still the drug of choice and will be drug of choice until introducing an alternative more effective than this agent.
